# Effects of Two Commercial Diets on Several Reproductive Parameters in Bitches: Note Two—Lactation and Puppies’ Performance

**DOI:** 10.3390/ani11010173

**Published:** 2021-01-13

**Authors:** Serena Calabrò, Alessandro Vastolo, Nadia Musco, Pietro Lombardi, Alessandro Troisi, Angela Polisca, Emanuela Vallesi, Riccardo Orlandi, Monica I. Cutrignelli

**Affiliations:** 1 Department of Veterinary Medicine and Animal Production, University of Napoli Federico II, 80137 Napoli, Italy; serena.calabro@unina.it (S.C.); alessandro.vastolo@unina.it (A.V.); pietro.lombardi@unina.it (P.L.); monica.cutrignelli@unina.it (M.I.C.); 2 School of Bioscience and Veterinary Medicine, University of Camerino, 62024 Matelica, Italy; alessandro.troisi@unicam.it; 3 Department of Veterinary Medicine, University of Perugia, 06124 Perugia, Italy; angela.polisca@unipg.it; 4 Tyrus Veterinary Clinic, Via A. Bartocci 1/G, 05100 Terni, Italy; manu0391@libero.it (E.V.); riccardo.orlandi83@hotmail.it (R.O.)

**Keywords:** dog, litter size, milk quality, growth dynamics, diet

## Abstract

**Simple Summary:**

Pregnancy dramatically changes the metabolic status of bitches. As a consequence, malnutrition negatively affects both reproductive parameters and survival rates of puppies. Therefore, before mating and conception, as well as during pregnancy and lactation, it is necessary to satisfy the bitches’ specific nutritional requirements. The present study aimed to compare the effects of two diets, Control (CTR) and Experimental (EX), which differ mainly as regards ingredients, energy, and protein contents, and which were administered to bitches of medium and large size from two months before the expected onset of proestrus up to 30 days after delivery. Bitches’ weight, body condition score, litter size, milk yield and quality, and puppies’ growth were evaluated. Considering the performance of the bitches and the puppies, it would seem that both the diets are adequate for bitches at these stages of their life cycle. In fact, after 30 days of lactation, all tested bitches showed a healthy status. Moreover, both the percentage of newborns mortality and puppies’ growth kinetics fell within the normal physiological range for the species.

**Abstract:**

The study aimed to compare two diets, Control (CTR) and Experimental (EX) (mainly differing as regards their ingredients, energy, and protein contents) administered to medium and large-sized bitches from two months before the expected proestrus and up to 30 days after delivery on mothers’ weight, body condition score, litter size, milk quality, and puppies’ growth. No differences were found for body weight during pregnancy, even if the BCS after delivery was significantly (*p* < 0.01) higher in the EX group than in the CTR one. Concerning the size effect, the percentage of weight gain on the initial body weight was double in medium-sized dogs compared to large dogs (*p* < 0.01). The number of puppies per litter was significantly higher (*p* < 0.05) for the EX group compared to the CTR one. Concerning puppies’ weight, the CTR group showed a significantly (*p* < 0.01) higher body weight from the 21st day of life due to the significant (*p* < 0.01) higher daily weight gain during the suckling period. Considering the performance of bitches and puppies, both diets seem useful for these stages of their lives. In fact, after 30 days of lactation, all tested bitches showed a healthy status and both the percentage of newborns mortality and puppies’ growth kinetics fell into the normal physiological range for the species.

## 1. Introduction

The impact of nutrition during pregnancy and early lactation on reproduction has been already recognized for some species [1,2]. By contrast, similar data is still missing for the canine species and several aspects of canine reproduction and fertility remain to be fully understood [3].

In all mammals, the metabolic status changes dramatically during pregnancy, and as a consequence, any mistake may negatively affect the reproductive parameters both in terms of deficiencies and excesses [3,4]. For these reasons, before mating and conception, it is necessary to guarantee the bitches ideal body weight and body condition score (BCS). Also, during both pregnancy and lactation, it is critical to satisfying the bitches nutritional requirements [5,6]. During estrus and up to the first two-thirds of pregnancy, the nutritional requirements are not higher than adult maintenance needs; indeed, the attention must be focused on bitches’ body weight and the quality of the administered diet in terms of nutrient digestibility and availability of micro and macro elements.

The assessment of a specific feeding plan for the bitches should be carried out routinely. In bitches, during the last three weeks of pregnancy, energy requirements for maintenance increase progressively from 1.25 to 1.50 times to sustain fetus development. Before and during pregnancy, certain macro- and micro-nutrients play specific roles in hormone production, placentation, and fetal development [2,7], thereby influencing newborns’ weight and survival [3]. Particular attention must be given to the essential fatty acids profile (linoleic, α-linolenic, arachidonic acids) and vitamins because these nutrients affect ovarian hormone production, uterine protein production, placentation, and fetal development [7]. In the last period of gestation, the protein requirements increase up to between 40–70% compared to the level required for maintenance [8]; a lack of proteins during pregnancy could negatively affect the puppies body weight at birth, mortality during the first 24 h of life, and may cause limited immunocompetence in puppies [9]. Puppies born from malnourished dogs have minor birth weight and often show low survival rates [6].

During lactation, the bitches’ daily energy requirements increase up to 2–4 times the levels required for maintenance [10,11,12], corresponding to about 145 kcal of digestible energy for a kg of metabolic weight [12]. Milk yield requirements were estimated [13] equal to 1200–1500 kcal/kg milk, taking into account also the stress related to caring for the puppies. During lactation, such requirements further increase in function of litter size. The milk produced is directly proportional to the number of puppies [9,12].

The lactation efficiency depends on the fat content in the diet. Increasing lipid percentage from 12 up to 20 on a dry matter basis (DM) causes the milk fat to increase by about 30% [14]. This latter nutrient represents the main energy source for newborns. As shown by Blanchard [15] in humans omega-3 fatty acids are necessary for fetal neurological development. Docosapentaenoic acid (DHA) during the last trimester of pregnancy and the first month of life, improves retina development, hearing, brain, and learning ability [16,17]. In dogs, as well as in humans, fetal enzymatic desaturation does not allow the achievement of adequate levels of polyunsaturated fatty acids [18]. For this reason, DHA is transferred before through the placenta to the fetus and later from maternal milk to the newborns.

The present investigation aimed to compare two diets (differing mainly as regards their ingredients, energy, and protein contents) administered to medium and large-sized bitches from two months before the expected onset of proestrus up to 30 days after delivery. Mothers’ weight, body condition score BCS, litter size, milk yield and quality, and puppies’ growth were monitored. We hypothesized that a specific diet, formulated with a higher concentration of macro and micro-nutrients might be able to improve bitches’ performance and puppies’ growth.

## 2. Materials and Methods 

All the procedures used in the study were approved (PG/2020/0044625) by the Ethical Animal Care and Use Committee of the University of Naples Federico II following local and national law regulations and guidelines (DL 26 March 4, 2014).

### 2.1. Feeding Protocol for Pregnant and Lactating Bitches

Twenty-two privately owned pluriparus 5 years old bitches were recruited for the study at a private veterinary clinic three months before the expected onset of proestrus and they were equally divided into two groups (Control_CTR and Experimental_EX). During the trial, 4 owners decided to leave the experimental protocol, consequently, we could follow only 18 bitches up to 30 days after delivery. The breeds of the dogs were as follows: Bernese Mountain dog (2), French Bulldog (2), English Bulldog (3), Dobermann (1), Argentine Dogo (2), Pitbull (1), Great Danes (1), Golden Retriever (1), Hovawart (1), Labrador Retriever (2), German Shepherd (1), Whippet (1). 

Taking into consideration their initial body weight, the groups were constituted as follows:

- Control group included 4 dogs of medium size (body weight <30 kg) and 5 of large size (body weight >30 kg) with a mean body weight after delivery equal to 32.6 ± 13.5 kg, mean BCS (5 points scale) equal to 2.7 ± 0.3.
- Experimental group consisted of 2 bitches of medium size and 7 of large size, with a mean body weight after delivery equal to 39.7 ± 14.8 kg mean BCS (5 points scale) equal to 3.4 ± 0.4.

Two diets differing as regards ingredients (starch and protein sources), energy, protein, and folate levels, named respectively CTR (ME 3933 kcal/kg DM) and EX (ME 4070 kcal/kg DM), were used (Table 1 and Table 2). Both diets were produced by Farmina Pet-foods (Nola, Italy); the first was a commercial kibble diet whereas the second was specifically formulated to perform the trial. The ingredients of the two diets are reported in Table 1. 

Both diets were supplemented with a mix of vitamins and microminerals in proportion to their specific energy level. In addition, both diets were supplemented with different levels of folate (CTR 215 and EX 220 μg/4000 kcal, CTR and EX, respectively).

Each group was fed one of the diets starting two months before the expected onset of proestrus until the puppies were weaned. During the trial, the daily individual rations were calculated according to NRC [19] indication, in the function of body weight and stage of pregnancy. From delivery up to puppies weaning, bitches were fed ad libitum.

### 2.2. Weight Control and Blood Analysis

The bitches were weighed the day after delivery and after 3 weeks of lactation when blood was collected from the cephalic vein and centrifuged at 1500× *g* for 20 min to obtain serum. 

Serum chemistry analyses were performed by an automatic biochemical analyzer AMS AUTOLAB (Rome, Italy) using reagents from Spinreact (Santa Colomna, Spain) to determine: blood urea nitrogen (BUN), creatinine (CREA), glucose (GLU) total proteins (TP), albumin (ALB), bilirubin (Bil T), aspartate aminotransferase (AST), alanine aminotransferase (ALT), gamma-glutamyltransferase (GGT), cholesterol (COL T) and triglycerides (TRI), chlorine (Cl) sodium (Na), calcium (Ca), and phosphorus (P).

Reactive oxygen metabolites (d-ROMs test) and biological antioxidant potential (BAP test) were also measured on serum aliquots using reagents from Diacron International s.r.l. (Grosseto, Italy) validated for canine species.

### 2.3. Milk Sampling and Analyses

After delivery, 15 mL of milk were collected three times (at days 7, 14, and 21 d) from different nipples. Milk samples were transported into pre-cooled boxes (3.0 ± 1.0 °C) to the Laboratory of Chemical analysis at the Department of Veterinary Medicine and Animal Production within 3.30 h of collection to determine fat and protein content (procedure 991.21 and 2000.18) [20].

### 2.4. Monitoring of Puppies 

The puppies were weighed daily by the owners during the first 10 days of life and then weekly until 30 days of age. From birth, puppies were clinically monitored every week until the end of weaning (60 days after birth) by checking heart rate, respiratory rate, rectal temperature, urinalysis, and fecal exams. 

### 2.5. Statistical Analysis

The data were analyzed by one-way ANOVA (JMP 14 software, SAS Institute, New York, NC, USA) according to the following model:

y_ijk_ = μ + D_i_ + S_j_ + G × S_ij_ + ε_ijk_ (1)

where y is the dependent variable, μ is the mean, D is the diet effect (CTR, EX), S is the dog size effect [medium (bodyweight < 30 kg) and large bodyweightt > 30 kg), G × S is the first level of interaction and ε is the error effect. For milk parameters, the sampling effect (S) was evaluated.

When significant differences were found in the ANOVA, means were compared using Tukey’s test.

Litter size data were analyzed using the Wilcoxon non-parametric test.

The correlations between serum and milk parameters and between milk composition and puppies’ performance were also evaluated (JMP 14 software, SAS Institute, New York, NC, USA).

## 3. Results

In Table 3, the variations in body weights and body condition scores (BCS, 5 points scale) of the bitches during pregnancy and lactation are depicted. No differences between groups were found for body weight during pregnancy even though the BCS after delivery was significantly (*p* < 0.01) higher in the EX group than in CTR one. During the first month of lactation, no differences in body weight or BCS were found between groups. 

Concerning the size effect, the percentage of weight gain on the initial body weight was double in medium-sized dogs compared to large-sized dogs (*p* < 0.01). No differences between size groups were shown for the body condition score. All the bitches showed an increase in body weight during pregnancy and a decrease in the first month of lactation.

The weight gain registered during pregnancy for medium size bitches was significantly higher (*p* < 0.01) than for larger dogs. The weight loss observed during lactation was higher (*p* < 0.01) for the bitches of the EX group which, after 30 days of milk production, showed a mean bodyweight lower than it initially was, whilst the LW_i_ of the CTR group was higher than it initially was (−0.54 vs. +4.36% LW_i_; for EX and CTR respectively, *p* < 0.01). On the other hand, the body condition score trend was quite similar for both treatments. 

Significant differences were observed for the number of puppies born per litter (litter size) (score mean: 9.11 vs. 3.89; *p* < 0.05 for EX and CTR one).

**Figure 1:**
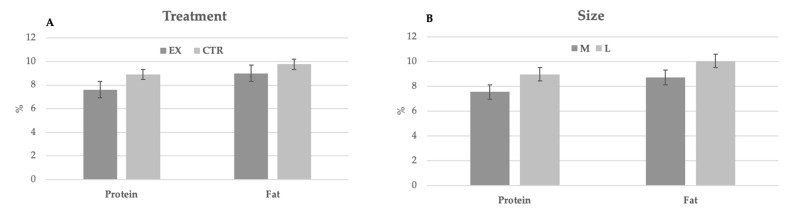
Milk chemical composition (% as fed, mean ± SD) of CTR group (n = 9) and EX group (n = 9) in function of the diets (**A**) and the size (**B**).

The main chemical characteristics of milk are reported in Table 4. No significant differences were observed between groups and dog size. During lactation, no significant differences were observed for both parameters in function of milk sampling. Nevertheless, the milk produced by larger bitches was richer in protein and fat than that of bitches of medium size (Figure 1).

**Figure 2:**
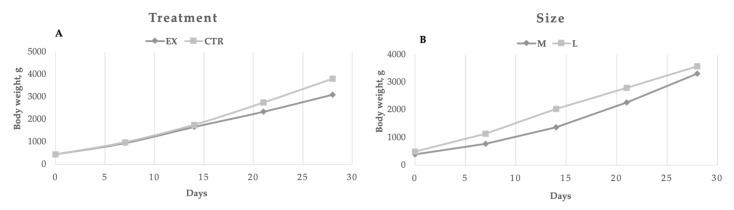
Puppies growth dynamic for CTR group (n = 42) and EX group (n = 67) in function of the diets (**A**) and the size (**B**).

Perinatal mortality (puppies died during the first week) was on average in the two groups equal to 6.8% (4.6 and 9.8% for the EX and CTR group, respectively). In Figure 2, the puppies’ performances are reported. At birth and during the first two weeks, the mothers’ dietary treatment did not affect puppies’ weight, whereas the CTR group showed a significantly (*p* < 0.01) higher bodyweight from the 21st day of age due to the significant (*p* < 0.01) higher values of daily weight gain during the suckling period 0–28 d (124.87 vs. 97.97 g/d) compared to EX group. Regarding the effect of dog size, significant differences (*p* < 0.01) were registered every week, except for the fourth one. However, the weight gain registered throughout the trial was similar for both large and medium-sized puppies. During the first week of life puppies of the EX group doubled their body weight, whereas CTR group puppies increased their weight by 1.39 times (*p* < 0.01). The dogs’ size did not affect the relative body weight gain during the first week of life (1.10 vs. 1.26 for medium and large dogs, respectively).

Concerning bitches’ biochemical parameters evaluated after 3 weeks of lactation (Table 5 and Table 6), only a few of them were affected by the treatment. More particularly, bitches fed the experimental diet showed significantly lower values of Ca*P and P (*p* < 0.05) than the control group. Medium-sized bitches were higher in Ca*P and P (*p* < 0.05) than the large-sized ones.

A significant correlation between serum parameters and milk composition was observed. More particularly, the percentage of milk fat was negatively related to serum glucose and cholesterol levels (r: −0.499; r: −0.550, respectively; *p* < 0.05), although the percentage of protein in the milk was not correlated with BUN of the bitches’ serum. Milk composition and, in particular fat percentage, was significantly and negatively correlated with puppies’ weight gain (r: −0.505; *p* < 0.05). Regarding blood parameters, Ca and d-ROMs levels were significantly related to puppies’ weight at 21 d (0.553 and 0.537, respectively; *p* < 0.05).

## 4. Discussion

Specific nutritional management is generally recommended during pregnancy and lactation. In this study, the owners of the recruited bitches were accustomed to using commercial balanced diets able to satisfy all nutritional requirements for adult dogs. 

Considering the performance of both bitches and puppies, both diets seemed suitable for these stages of life. In fact, after 30 days of lactation, all tested bitches showed a healthy status and both the percentage of newborns mortality and puppies’ growth kinetics fell in the physiological range for the species. In any case, the higher litter size registered in the experimental group seemed to confirm that the diet, richer in crude protein, essential fatty acids, vitamins, and minerals, allowed a better embryonal development as observed in a previous note where the results obtained during pregnancy of this trial are reported [21].

Importantly, all bitches did not gain too much bodyweight during pregnancy (no more than 15–25% of initial weight in function of litter size), thus limiting excessive weight at the end of pregnancy (into the range of 5–10%) indicated as ideal by Greco [7]. On the other hand, excessive weight loss during lactation, due to the negative energy balance that may occur after delivery [22], could represent a metabolic risk [23]. Indeed, lactation is the stage in a dog’s life during which energy deficiencies are immediately evident as weight loss and BCS reduction [24]. The higher weight loss during lactation registered in the EX group could be explained by the larger litter sizes, which probably resulted in a higher milk yield considering that milk production is related to the number of puppies at litter [19]. The use of different carbohydrate sources at this stage could have affected the availability of energy due to differing post-prandial glucose responses [25,26]. Moreover, it is interesting to underline the differences in relative weight gain registered between large and medium-sized bitches that might suggest a different nutrient utilization in bitches of different sizes. 

Must it be taken into account that the use in the trial of different dog breeds, instead of a specific breed, could represent a limit for the study. Nevertheless, the use of a cohort of dogs heterogeneous, in terms of size and breeds, could reveal general effects not segregated on race specificity. Middelton et al. [27], evaluating 449 metabolites and 16 clinic parameters in 83 dogs of different breeds, observed significant differences between small and large dog sizes. In particular, large size dogs showed higher antioxidant status and differences in circulating amino acids. The authors suggested that these differences could be ascribable to the dissimilar intestinal microbiota or different catabolic pathways. 

Our data underline some significant variations in milk composition during the suckling period. Regardless of the nutritional treatment, the large-sized bitches showed higher milk protein and fat contents throughout the lactation period. Only a few data is available regarding the composition of dog milk. Our results are in contrast with those reported by Lönnerdal et al. [28] who found in beagles lower protein contents and a significant increase in milk protein percentage during lactation (from 4.30 to 5.31% at 10 and 30 days of lactation, respectively). In German shepherd milk, Dokoupilová et al. [29] reported a protein concentration varying from 7.08 to 7.51% from the 7th to 30th day of lactation. Similarly, to our results, Oftedal [30] reported a mean protein and fat concentration equal to 7.53 and 9.47%, respectively. Several authors have reported a non-significant increase in fat content from day 10 to day 25 in German shepherds [29,31] and beagles [32]. These authors have suggested that the variations between studies may represent the actual differences between dog breeds and other factors, such as litter size and the characteristics (composition, energy intake micro- and micro-nutrients availability) of the diets administered to the bitches could probably also affect these results. In contrast, Russe [33] reported only slight changes in milk composition among breeds. 

The performance registered in puppies (e.g., perinatal mortality, body weight, and daily weight gain) during the first month are in line with the range indicated by Fascetti [23] who suggested a mean daily weight gain of 2–4 g/day/kg of adult weight for the first 5 months of life as correct growth performance, with the highest growth rate during the first month. 

No statistical differences were detected between the groups as regards all biochemical parameters, thus suggesting that both diets were well tolerated. The same can be said as regards the evaluation of oxidative status. The d-ROMs levels observed in both groups fell in the normal range values for the canine species proposed by Pasquini et al. [34] and by Sechi et al. [35]; no significant differences were seen between groups. These results could be due to the supplementation of molecules with an antioxidant role, such as tocopherols and selenium used for both diets [36].

Folate supplementation is recommended in humans during pregnancy to prevent malformation, particularly neural tube defects. Elwood & Colquhoun [37] reported, in Boston terriers, a decrease in the percentage of cleft palates in puppies fed a diet supplemented with 5.0 mg/day of folate. The potential benefit of folate supplementation at the same dose was shown by administering it to French bulldog bitches from 15 days before mating until the end of the gestation period; a clear reduction (48.54%) in cleft palates was observed [38]. The occurrence of lip and/or palate cleft in newborns puppies of two breeds, pugs and chihuahuas, was reduced by d by administering a folate daily dose of 5.0 mg per pug and 2.5 mg per chihuahua from the onset of heat till up to the 40th day of gestation, as reported Domosławska et al. [39]. As observed in the reported studies, folate supplementation in bitches, as well as in women, is not able to prevent all cases of cleft palate, probably because of the multifactorial nature of such disorder. Although no adverse effects of feeding folate have been demonstrated, the NRC system [19] advises not to exceed 1000 times the conventional recommendation of 0.18 mg/kg of dry matter. Indeed, despite the different levels of folate supplementation between the two groups, no cases of cleft palate were observed in our study, suggesting that the standard folate supplementation of the CTR diet was sufficient to guarantee the animals’ health.

The higher level of Ca/P and phosphorus registered in the control group suggests that the higher concentration of calcium and phosphorus in the experimental diet was not sufficient to satisfy the bitches requirement, although these bitches may have compensated for this shortage with their organic reserve during lactation. Usually, breeders and practitioners tend to over-supplement bitches’ diets during gestation and lactation with different minerals, such as calcium. This practice could be inappropriate for the bitches’ health. Even if the Ca requirement at these stages could be higher than adult maintenance ones, oversupplying Ca is not beneficial: when the Ca administered from the beginning of pregnancy is over supplemented, serum calcium concentration remains high and results in a down-regulation of parathyroid activity [40]. It is well known that calcium homeostasis is mainly regulated by this hormone that promotes calcium mobilization from bones [37]. It also is known that during lactation, as well as during gestation, various mammalian species use their bone mineral deposits in addition to the oral supply of calcium and phosphorus to meet the newborns growing demand. Beagle studies have shown that an increase in intracortical bone remodeling occurs during breastfeeding [41,42]. The ratio between calcium and phosphorus, rather than the Ca level in the diet administered during pregnancy, plays a pivotal role in causing eclampsia. The correlation between calcium and phosphorus intake and digestibility and their influence on serum parameters and bone turnover has been poorly studied. A loss of stabilized calcium linked to the membranes leads to their greater permeability, which results in them being more easily depolarized. It is essential to ensure sufficient calcium and phosphorus intake to prevent this condition and to avoid excessive bone resorption.

This mechanism could explain the similar levels of serum calcium in EX and CTR groups, despite the higher Ca concentration in the EX diet than the CTR ones. Further studies are needed to investigate the causes of the increase in serum Ca/P and P levels observed in the dogs in the EX group.

In any case, the significant correlation between bitches’ serum calcium level and puppies’ weight gain observed in this trial seems to confirm the role of this mineral in healthy fetal development.

## 5. Conclusions

In conclusion, the experimental diet seemed to guarantee higher performance in terms of litter size and correct weight gain during pregnancy than the control one. Nevertheless, milk composition and weight loss after 30 d of lactation were more favorable for the control group. This data is probably due to the higher milk yield necessary to maintain the growth of a higher number of puppies in the experimental group. This hypothesis seems confirmed by the higher relative weight gain registered during the first week of life in the CTR group. The supplementation in the EX diet, mainly regarding Ca, P, DHA, EPA, and folate did not lead to significant health differences in bitches or puppies. Importantly, the EX diet failed to improve the oxidative status thus suggesting that different doses or substances should be tested to achieve such a goal. Moreover, Ca and P supplementation did not optimize their serum levels, suggesting that the influence of these two minerals intake on mineral metabolism in bitches needs to be further studied.

## 6. Limitations

This study has some limitations: (1) the use in the trial of different dog breeds instead of a specific dog breed; (2) the categorization of dogs’ size only in terms of body weight (>30 kg and <30 kg); (3) grouping of breeds into CTR and EX groups it’s not completely balanced; (4) the limited sample size (9 dogs per group).

## Figures and Tables

**Table 1:** Diet ingredients.

Source of	CTR	EX
Protein	Poultry meal, fishmeal	Poultry meal, fishmeal, dehydrated eggs
Carbohydrate	Rice, corn, beet pulp	Spelt, oat, potato, beet pulp
Lipid	Poultry fat, fish oil, seed oil	Fish oil, flaxseed
Supplementation	Sodium chloride, dehydrated *Saccharomyces cerevisiae*	Calcium carbonate, monocalcium phosphate, potassium chloride, psyllium, MOS, sodium chloride, dehydrated *Saccharomyces cerevisiae*

**Table 2:** Characteristics of the diets Control (CTR) and Experimental (EX) administered to the bitches during the experimental period.

Diets Characteristics	Unit	CTR (3933 kcal/kg DM))	EX (4070 kcal/kg DM)
Crude protein	g/4000 kcal	299.9	330.5
Ether extract	g/4000 kcal	188.6	188.9
Crude fiber	g/4000 kcal	23.90	33.09
Ash	g/4000 kcal	77.96	93.27
Ca	g/4000 kcal	4.35	8.40
P	g/4000 kcal	3.79	8.39
Mg	g/4000 kcal	0.601	0.612
Methionine	g/4000 kcal	3.799	4.124
Cysteine	g/4000 kcal	2.521	3.360
Threonine	g/4000 kcal	4.818	5.393
Lysine	g/4000 kcal	3.933	4.477
Tryptophan	g/4000 kcal	1.573	1.750
Linoleic acid	g/4000 kcal	12.01	13.50
α-linolenic acid	g/4000 kcal	0.407	0.822
Arachidonic acid	mg/4000 kcal	0.280	0.300
EPA + DHA	g/4000 kcal	0.467	0.510

**Table 3:** Trend of bitches’ body weight (LW) and body condition score (BCS) during pregnancy and lactation.

Group	LW_i_	LW_f_	LW_l_	BCS_i_	BCS_f_	BCS_l_	ΔLW_P_	ΔLW_l_
	kg			5-Point Scale			% LW_i_	
CTR	27.29	32.56	28.48	3.29	3.55	2.83	+19.31	+4.36 ^A^
EX	33.16	39.73	32.98	3.62	3.42	2.92	+19.81	−0.54 ^B^
M size	19.26 ^B^	24.97 ^B^	20.47 ^B^	3.46	3.08	3.00	+29.64 ^A^	+6.28 ^A^
L size	41.19 ^A^	47.32 ^A^	42.00 ^A^	3.46	3.08	2.75	+14.88 ^B^	+1.97 ^B^
Interaction	NS	NS	NS	NS	NS	NS	NS	NS
MSE	75.44	82.77	79.43	0.17	0.12	0.07	44.38	7.21

EX: experimental; CTR: control; M: medium; L: large; LW_i_: initial body weight; LW_f_: body weight after delivery; LW_l_: body weight at the end of lactation; BCS_i_: initial Body condition score; BCS_f_: body condition score after delivery; BCS_l_: body condition score at the end of lactation; ΔLW_P_: variation of body weight for pregnancy = (LW_f_−LW_i_/LW_i_)*100; ΔLW_l_: variation of body weight for lactation = (LW_l_−LW_i_/LW_i_)*100. NS: not significant; MSE: A, B: *p* < 0.01.

**Table 4:** Milk chemical composition (% as fed, mean ± SD) in the two groups along the trial.

Group	CTR		EX	
Sampling	Protein	Fat	Protein	Fat
1	8.85 ± 1.09	10.21 ± 1.19	7.55 ± 1.10	8.47 ± 1.21
2	8.98 ± 1.03	9.51 ± 1.35	7.68 ± 0.96	9.25 ± 1.05
3	8.92 ± 1.03	9.93 ± 1.13	7.61 ± 1.04	9.18 ± 1.14
**Size**	**Medium**		**Large**	
Sampling	Protein	Fat	Protein	Fat
1	7.49 ± 1.09	8.31 ± 1.02	8.92 ± 1.10	9.67 ± 1.20
2	7.61 ± 1.00	8.96 ± 1.10	9.04 ± 0.99	10.29 ± 1.08
3	7.55 ± 0.98	8.89 ± 1.13	8.98 ± 1.04	10.22 ± 1.14

CTR: control; EX: experimental; M: medium; L: large. Sampling 1, 2, and 3: 7, 14, and 21 days of lactation, respectively.

**Table 5:** Effect of treatment and size on bitches’ hematic profile during the trial.

Hematic Profile	BUN	Crea	Glu	TP	Alb	Bil T	AST	ALT	GGT	PA	COL T	TRI	d-ROMs	BAP
Group	mg/dL	mg/dL	mg/dL	g/dL	g/dL	g/dL	Ul/L	Ul/L	Ul/L	Ul/L	mg/dL	mg/dL	UCARR	μmoles/L
*Treatment*														
CTR	80.7	1.23	137	6.45	3.92	0.77	33.5	21.3	3.90	138	320	107	169.83	4102.17
EX	59.7	1.65	123	6.25	3.57	0.46	37.0	28.0	3.25	146	228	74.7	180.50	3878.50
*Size*														
M size	86.0	1.42	131	6.13	3.98	0.78	30.2	19.7	3.42	171	328	102	161.67	4622.63
L size	54.5	1.46	129	6.55	3.51	0.45	40.2	29.7	3.72	114	221	79.0	188.67	3357.83
MSE	144	0.05	25.7	0.16	0.23	0.05	60.5	60.3	0.60	399	2532	442	69.55	568.12

CTR: control; EX: experimental; M: medium; L: large; Bil T: total bilirubin; TRI: triglycerides; COL T: total cholesterol; TP: total protein; MSE: mean square error.

**Table 6:** Effect of treatment and size on bitches’ mineral profile during the trial.

Mineral Profile	Cl	Na	K	Na/K	Ca	Ca*P	P
Group	mEq/L	mEq/L	mEq/L		mg/dL		mg/dL
*Treatment*							
CTR	117	156	4.40	33.9	13.1	106 ^a^	8.34 ^a^
EX	123	156	4.60	34.2	12.5	72.5 ^b^	5.71 ^b^
*Size*							
M size	120	159	4.90	31.9	13.7	113 ^a^	8.51 ^a^
L size	121	153	4.10	36.3	11.8	65.9 ^b^	5.54 ^b^
MSE	3.55	81.7	0.19	5.33	0.27	117.8	0.44

CTR: control; EX: experimental; M: medium; L: large; Cl: chlorine; Na: sodium; K: potassium; Ca: calcium; Ca*P: calcium*phosphorus; P: phosphorus. MSE: mean square error; a, b: *p* < 0.05.

## Data Availability

The data presented in this study are available on request from the corresponding author.
